# Integration of minimally invasive techniques and interventional therapy: application of percutaneous nephrolithotomy in patients with upper urinary tract stones and an analysis of risk factors for postoperative bleeding

**DOI:** 10.3389/fmed.2025.1556224

**Published:** 2025-05-02

**Authors:** Hao Sun, Zhong Chen

**Affiliations:** ^1^Department of Urology, Zibo 148 Hospital, Zibo, Shandong, China; ^2^Department of Interventional Vascular, Zibo 148 Hospital, Zibo, Shandong, China

**Keywords:** percutaneous nephrolithotomy, upper urinary tract stone, postoperative bleeding, guidewire-assisted, risk factors

## Abstract

**Background:**

Percutaneous nephrolithotomy is one of the preferred treatment options for upper urinary tract stones. However, postoperative bleeding remains a clinical challenge. It is crucial to identify the effectiveness of this procedure and understand the risk factors causing postoperative bleeding.

**Methods:**

A total of 383 patients with upper urinary tract stones included in our hospital from March 2020 to February 2024 were retrospectively selected and divided into 2 groups as per different treatments. A total of 204 patients who underwent guidewire-assisted percutaneous nephrolithotomy were included in the guidewire-assisted group, while the other 179 patients who underwent conventional percutaneous nephrolithotomy were enrolled in the conventional group for a comparison of treatment effects. Then, single-factor and multifactorial logistic regressions in accordance with the postoperative bleeding situation were conducted to analyze the risk factors of postoperative bleeding in patients with upper urinary tract stones.

**Results:**

The results showed that the guidewire-assisted percutaneous nephrolithotomy group had a higher stone removal rate compared to the conventional group, with lower rates of complications, operation time, gastrointestinal recovery time, hospital stay, postoperative bleeding, and hemoglobin drop (*p* < 0.05). There was no significant difference in stone recurrence rate (*p* > 0.05). Among the 383 patients studied, 39 experienced severe bleeding (≥400 mL), while 344 had minor bleeding (<400 mL). Factors significantly associated with postoperative bleeding included the history of diabetes, preoperative blood creatinine, surgical method, staghorn calculi, hydronephrosis, and renal parenchymal thickness difference (*p* < 0.05). However, collinearity was noted between diabetes history and staghorn calculi. After adjustment of these variables, preoperative blood creatinine, surgical modality, hydronephrosis, and renal parenchymal thickness emerged as key predictors of postoperative bleeding.

**Conclusion:**

Compared to conventional percutaneous nephrolithotomy, guidewire-assisted percutaneous nephrolithotomy could improve the stone removal rate of patients with upper urinary tract stones and reduce the occurrence of complications, while some patients were still prone to the postoperative bleeding phenomenon, which might be closely related to the preoperative Scr, surgical methods, hydronephrosis, and renal parenchymal thickness. The mentioned phenomenon needed clinical attention and corresponding measures to intervene as soon as possible to reduce the bleeding in the postoperative period.

## 1 Introduction

Upper urinary tract stones, one of the more common urinary system diseases, typically occur in the kidney or upper ureter. In recent years, with the rapid social and economic development and changes in dietary structure and habits, the incidence of this condition has been steadily increasing (1). According to the statistics (2), the worldwide morbidity of upper urinary tract stones is approximately 5%–10%. It has also been reported (3) that the prevalence of upper urinary tract stones is relatively low in Asia, usually ranging from 1% to 5%. If effective treatment is not taken in time, as the condition continues to aggravate, it will easily lead to severe hydronephrosis and uremia, which will directly endanger the patient’s life. Although conservative measures often taken in the past can reduce relevant symptoms, the long-term effect is not ideal with a high recurrence rate, which is not conducive to disease recovery (4). Surgery has become the first choice for this disease with a high success rate, especially with the development of ureteroscopy and nephrolithotomy technology in recent years, urinary stone treatment has shifted from traditional open surgical treatment to minimally invasive laparoscopic technology. Studies have found (5) that guidewire assistance in percutaneous nephrolithotomy as an auxiliary tool for dilating the ureter and renal channel can be used along the guidewire by a dilation kit to gradually dilate the ureter and renal channel so that the nephrolithotomy can enter the kidney smoothly and play a supporting role during the ureteral dilation process to maintain the stability of the channel, as well as reduce the difficulty of puncture and the risk of bleeding. Compared to conventional percutaneous nephrolithotomy, it boasts the advantages of low trauma, high efficiency, and fast recovery. It effectively avoids tissue damage and bleeding induced by improper operation, sharply alleviates postoperative bleeding, significantly improves the stone removal rate, and promotes rapid postoperative recovery.

However, Singh et al. (6) stated that bleeding was the most common and serious complication after percutaneous nephrolithotomy, with its incidence reaching approximately 7% to 20%. Another report suggested (7) that the renal blood supply was abundant, with an average of approximately 200 mL of blood flowing through both kidneys per minute. Improper or delayed treatment can lead to serious consequences and may even endanger the patient’s life. Therefore, it is crucial to identify the risk factors for postoperative bleeding at an early stage. By identifying risk factors, clinicians are able to better assess postoperative bleeding risks in patients and take appropriate preventive measures, such as adjusting the surgical strategy, optimizing preoperative preparations, and strengthening postoperative monitoring, thus substantially reducing the incidence and severity of postoperative bleeding (8). Notably, guidewire-assisted percutaneous nephrolithotomy is usually considered to reduce postoperative bleeding (9), and a previous study indicated that postoperative bleeding after guidewire-assisted percutaneous nephrolithotomy was significantly less than that of conventional percutaneous nephrolithotomy. In addition, the chance of severe bleeding after guidewire-assisted percutaneous nephrolithotomy is only 1.06%, whereas the incidence of severe bleeding after conventional percutaneous nephrolithotomy reaches 5.13%. Therefore, it can be demonstrated that the risk of bleeding should be a key clinical consideration when choosing a surgical procedure.

Clinical reports are limited about the application of percutaneous nephrolithotomy in patients with upper urinary tract stones and the risk factors for postoperative bleeding at this stage. Therefore, this study carries out an in-depth and systematic study by analyzing the effect of this procedure in the treatment of patients with upper urinary tract stones, which provides clinicians with a more accurate and effective treatment plan, improves the success rate of the operation and the removal rate of the stones, and reduces the incidence of complications. Together with a detailed discussion of the risk factors for postoperative bleeding, we render a more precise basis for predicting and preventing postoperative bleeding.

## 2 Methods

### 2.1 Research object

Sample size calculation formula: *n* = [(Z_α/2_)^2^ × *p*(1 − *p*)] ÷ E^2^, where *n* represents the sample size, *Z_α/2_* is the Z-value corresponding to the significance level, *p* is the population proportion, and *E* is the margin of sampling error. According to this formula, at least 383 patients should be selected to ensure that the sampling error of the estimated population proportion does not exceed 0.05 with a 95% confidence level. Therefore, this study retrospectively analyzed 383 patients with upper urinary tract stones who were admitted to our hospital from March 2020 to February 2024. Among them, 204 patients who underwent guidewire-assisted percutaneous nephrolithotomy were included in the guidewire-assisted group, while 179 patients who underwent conventional percutaneous nephrolithotomy were included in the conventional group. The study was approved by the Ethics Committee of Zibo 148 Hospital (ZB148H-LL-LC20240101), and the relevant provisions of *the Declaration of Helsinki* were strictly observed.

### 2.2 Inclusion and exclusion criteria

Inclusion criteria: (1) complete clinical information and follow-up data; (2) diagnosed by urological CT, ultrasound, and other examinations; (3) met the indications for percutaneous nephrolithotomy; (4) clear cognition and consciousness and actively cooperated with the completion of the examinations and surgery; and (5) no urological infection or the infection had been controlled.

Exclusion criteria: (1) the presence of other urological diseases, (2) contraindications to surgery or anesthesia, (3) pregnant or breastfeeding women, (4) those with combined metabolic dysfunction disorders or hematological disease, and (5) urethral or ureteral stenosis or obstruction.

### 2.3 Flowchart

**Figure 1:**
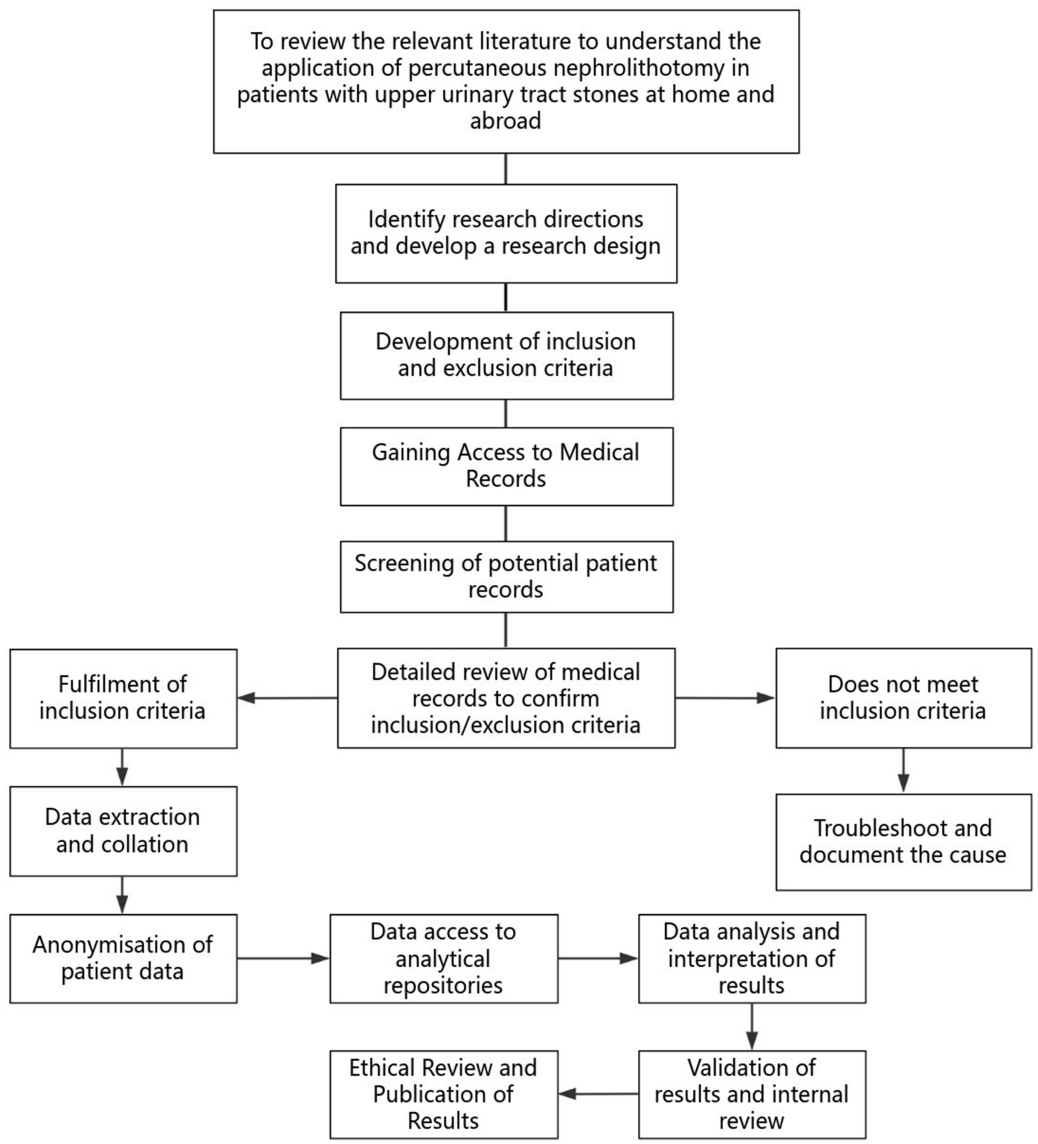
Flowchart.

The flowchart illustrates the patient selection process for the study (Figure 1).

### 2.4 General situation

Based on the hospital information management system, clinical data of patients with upper urinary tract stones who met the inclusion and exclusion criteria were collected regarding age, gender, body mass index, disease duration, stone diameter, stone location, number of stones, history of hypertension (in accordance with the diagnostic criteria for hypertension in *The Japanese Society of Hypertension Guidelines for Self-monitoring of Blood Pressure at Home (Second Edition)*) (10), history of diabetes mellitus (in accordance with the diagnostic criteria for diabetes mellitus in the *Application of the Chinese Expert Consensus on Diabetes in clinical practice*) (11), and urinary tract infection (mid-urine sediment leukocyte count > 10 HP/L was considered urinary tract infection).

### 2.5 Imaging examination

Philips Brilliance 16CT was used for low-dose scanning, with a voltage of 120 kV, a current of 150 mA, a layer thickness of 10 mm, and a pitch of 1.0. The patient lay flat on the scan table in the supine position, and the scan was completed in a single breath-hold, with the observation of the stone diameter, the location of the stones, the number of stones, and the morphological characteristics of the stones (staghorn calculi: similar to the shape of deer’s antler). The thickness of the renal parenchyma in the ventral, lateral, and dorsal regions of the upper, middle, and lower calyces was selected, and the average of the thickness of the renal parenchyma at each location was taken as the thickness of the renal parenchyma. If the image of the affected side showed a non-echoic area and the wall of the capsule was smooth, it was considered the affected side of the renal cyst.

### 2.6 Laboratory examination

Preoperatively, 3 mL of venous blood was collected from all patients, centrifuged at 3,000 r/min (8 cm radius), and the supernatant was taken after 10 min for examination. White blood cell count (WBC), neutrophil count (NEUT), and hemoglobin level were detected by a fully automatic blood cell analyzer (Myriad BC-5000,China). Blood creatinine (Scr) was measured using a fully automatic biochemical analyzer (Olympus, Au2700, Japan) (renal insufficiency was defined if Scr was elevated to more than 1.5 times the normal value).

### 2.7 Hydronephrosis

No hydronephrosis: no dilatation of the collecting system and intact kidney calyces(12); mild hydronephrosis: no obvious abnormality in the morphology and size of the kidneys, normal thickness and echogenicity of the renal parenchyma and renal collecting system separation of 2–3 cm; moderate hydronephrosis: mild increase in renal volume, fullness of the morphology, mild thinning of the parenchyma, unclear display of the renal columns, obvious dilatation of the renal pelvis and kidney calyces and renal collecting system separation of 3–4 cm; severe hydronephrosis: increased kidney volume with abnormal morphology, invisible or remarkable thinning of the parenchyma, liquid dark area in whole renal area.

### 2.8 Operative method

The conventional group: Conventional percutaneous nephrolithotomy was performed with the patient in the prone position and under general anesthesia. The F18 fascial dilator with sheath was inserted into the kidney through a skin incision to bring the holmium laser optical fiber inside, thus accurately crushing the stone. During the lithotripsy process, the physician needed to carefully control the power and frequency of the holmium laser to break up the stones gradually into smaller particles so that they could be naturally eliminated from the body via the urinary tract.

The guidewire-assisted group: Guidewire-assisted percutaneous nephrolithotomy was executed with general anesthesia and position adjustment according to the needs of the operation, first in the cystotomy position, then inserting a ureteral catheter into the ureteral stone on the affected side, changing the patient’s position to prone, and placing a soft pillow under the abdomen to elevate the lumbar region. The location of the puncture was determined by ultrasound guidance, and the point of puncture was set between the 11th and 12th ribs under the subscapularis line on the affected side. After a successful puncture, a Zebra guidewire was imported, and a stone extraction channel was established by a fascial dilator to dilate the channel. The nephrolithotomy was inserted into the kidney to confirm the location of the stone, and then broke the larger stone by pneumatic ballistic lithotripsy and flushed out the stone to remove it completely. After lithotripsy, the nephrolithotomy was withdrawn and a double J-tube was inserted, which was removed 1 month after the operation.

### 2.9 Postoperative bleeding

After percutaneous nephrolithotomy, all patients were observed for bleeding, which was quantitatively measured and visually estimated (13), of which ≥400 mL was defined as severe bleeding, while <400 mL was determined as a small amount of bleeding.

### 2.10 Observation target

After both groups received surgical treatment, operation time, postoperative bleeding, hemoglobin drops (the D-value of the pre- and post-operative hemoglobin on the first day), gastrointestinal function recovery time, and hospital days were observed. The rate of stone removal (the largest diameter of the residual stone registered < 4 mm, which was considered to be the removal of stones), the rate of complications (including infection, fever, bleeding, and urinary extravasation), and the stone recurrence rate (recurrence was defined as the reappearance of a new stone at the primary lesion) were counted through outpatient follow-up (supplemented by telephone follow-up).

### 2.11 Statistical analysis

In this paper, data were calculated by SPSS 25.0 statistical software with count data expressed as percentages and the *χ^2^* test adopted; measurements that conformed to the normal distribution were expressed as ( $\bar{\chi \;}$ ± s) with the *t-test* introduced, and those that did not conform were expressed by the median and interquartile spacing [M(P_25_, P_75_)]. Influences on statistical differences in the univariate analysis were incorporated into the logistic regressions to perform analysis with a test level of *α* = 0.05. A *p*-value of <0.05 referred to statistical significance.

## 3 Results

### 3.1 Comparison of clinical data between groups

A comparison of the clinical data between the two groups revealed that there was no significant difference between the guidewire-assisted group and the conventional group in terms of age, gender, body mass index, duration of disease, stone diameter, stone location, number of stones, history of hypertension, history of diabetes mellitus, and urinary tract infections (*p* > 0.05). The details are shown in Table 1.

**Table 1:** Comparison of clinical data between groups (*n* = 383).

Clinical data		*n*	Guidewire-assisted group (*n* = 204)	Conventional group (*n* = 179)	*χ^2^*/*Z*	*p*
Age [Year, M(P_25_, P_75_)]		383	52.00 (48.00,57.80)	52.00 (47.00,56.00)	−1.215	0.224
Sex (*n*, example)	Male	214	118	96	0.686	0.408
	Female	169	86	83		
Body mass index [kg/m^2^, M(P_25_, P_75_)]		383	22.10 (21.20,23.10)	21.90 (20.90,23.00)	−1.819	0.069
Duration of disease [Year, M(P_25_, P_75_)]		383	2.50 (2.00,3.00)	2.00 (2.00,3.00)	−1.628	0.103
Stone diameter [mm, M(P_25_, P_75_)]		383	2.95 (2.50,3.70)	3.30 (2.60,3.90)	−1.884	0.060
Stone location (*n*, example)	Upper and middle calyces stones	210	118	92	1.600	0.206
	Lower calyces stones	173	86	87		
Number of stones (*n*, example)	Single	226	125	101	0.927	0.336
	Multiple	157	79	78		
History of hypertension (*n*, example)	Yes	117	64	53	0.140	0.709
	No	266	140	126		
History of diabetes mellitus (*n*, example)	Yes	110	61	49	0.298	0.585
	No	273	143	130		
Urinary tract infections (*n*, example)	Yes	85	48	37	0.451	0.502
	No	298	156	142		

### 3.2 Comparison of treatment outcomes between groups

Comparing the treatment effects of the two groups, it was found that the stone clearance rate of the guidewire-assisted group (92.16% vs. 84.36%) was higher than that of the conventional group, while the complication rate (25.49% vs. 35.20%) was lower than that of the conventional group (*p* < 0.05). There was no statistically significant difference in the recurrence rate of stones between the guidewire-assisted group and the conventional group (5.88% vs. 10.61%) (*p* > 0.05, Table 2).

**Table 2:** Comparison of treatment outcomes between groups [*n* (%)].

Groups	Stone clearance rate	Complication rate	Recurrence rate
The guidewire-assisted group (*n* = 204)	188 (92.16)	52 (25.49)	12 (5.88)
The conventional group (*n* = 179)	151 (84.36)	63 (35.20)	19 (10.61)
*χ^2^*	5.704	4.274	2.870
*p*	0.017	0.039	0.090

### 3.3 Comparison of perioperative indicators between groups

**Figure 2:**
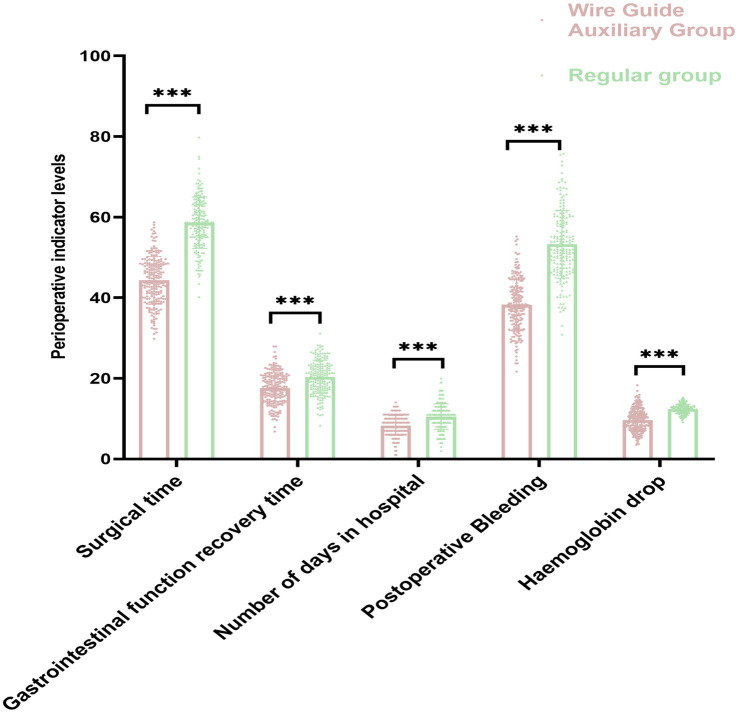
Comparison of perioperative indicators between groups (^***^ indicates statistical significance).

Comparing the perioperative conditions of the two groups, it was detected that the operation time (44.31 ± 5.92) min, gastrointestinal function recovery time (17.56 ± 3.84) h and hospital stay (8.31 ± 2.47) d in the guidewire-assisted group were shorter than those in the conventional group (58.62 ± 6.34) min, (20.39 ± 4.11) h, and (10.52 ± 3.15) d. At the same time, compared to the postoperative bleeding (53.26 ± 8.39) and hemoglobin drop (12.44 ± 1.16) in the conventional group, the postoperative bleeding (38.17 ± 6.32) and hemoglobin drop (9.65 ± 2.83) in the guidewire-assisted group were lower (*p* < 0.05, Figure 2).

### 3.4 Unifactorial analysis of bleeding after percutaneous nephrolithotomy

In this paper, a total of 383 patients with upper urinary tract stones underwent percutaneous nephrolithotomy to observe postoperative bleeding, of which 39 patients (10.18%) with bleeding volume ≥ 400 mL were included in the severe bleeding group, and the remaining 344 patients (89.82%) with bleeding volume < 400 mL were included in the mild bleeding group. Comparison of the two groups mentioned revealed significant differences in terms of history of diabetes mellitus, history of hypertension, preoperative Scr, surgical approach, staghorn calculi, hydronephrosis, and renal parenchymal thickness (*p* < 0.05, Table 3).

**Table 3:** Unifactorial analysis of bleeding after percutaneous nephrolithotomy (*n* = 383).

Clinical data		*n*	Severe bleeding group (*n* = 39)	Mild bleeding group (*n* = 344)	*χ^2^*/*t*/*Z*	*p*
History of diabetes mellitus (*n*, example)	Yes	110	26	84	30.541	<0.001
	No	273	13	260		
History of hypertension (*n*, example)	Yes	117	16	101	2.247	0.134
	No	266	23	243		
Preoperative WBC (×10^9^/L)		383	6.54 ± 2.08	6.28 ± 2.14	0.721	0.471
Preoperative NEUT (×10^9^/L)		383	8.37 ± 2.69	8.46 ± 2.72	0.196	0.845
Preoperative Scr[μmol/L, M(P_25_, P_75_)]		383	123.50 (117.90,132.50)	104.65 (98.40,109.40)	−8.453	<0.001
Surgical approach (*n*, example)	Guidewire-assisted percutaneous nephrolithotomy	204	12	192	8.826	0.003
	Conventional percutaneous nephrolithotomy	179	27	152		
Staghorn calculi (*n*, example)	Yes	120	23	97	15.422	<0.001
	No	263	16	247		
Hydronephrosis (*n*, example)	No/Mild	217	14	203	7.621	0.006
	Moderate/Severe	166	25	141		
Renal parenchymal thickness [mm, M(P_25_, P_75_)]		383	12.00 (10.90,13.20)	8.70 (6.90,10.20)	−7.827	<0.001
Kidney cysts on the affected side (*n*, example)	Yes	13	2	11	0.398	0.528
	No	370	37	333		
Renal insufficiency (*n*, example)	Yes	40	5	35	0.262	0.609
	No	343	34	309		

### 3.5 Assignment of research variables and covariance analysis

Bleeding after percutaneous nephrolithotomy was taken as the dependent variable, and history of diabetes mellitus, preoperative Scr, surgical procedure, staghorn calculi, hydronephrosis, and renal parenchymal thickness were taken as independent variables X1, X2, X3, X4, X5, and X6 with corresponding assignments carried out for each of the independent variables, respectively (Table 4). Upon the diagnosis of covariance, the VIF values of the history of diabetes and staghorn-shaped stone were >5, indicating that the item had a covariance problem, which could be considered to be moved out of the model for secondary analysis. The VIF values of preoperative Scr, surgical procedure, hydronephrosis, and renal parenchymal thickness registered <5, suggesting no covariance problem, which could be calculated by substituting them into the logistic prediction model. The details are shown in Table 5.

**Table 4:** Assignment of research variables.

Variables	Factors	Assignment
Y	Bleeding after percutaneous nephrolithotomy	0 = hemorrhage < 400 mL, 1 = hemorrhage ≥ 400 mL
X1	History of diabetes mellitus	0 = no, 1 = yes
X2	Preoperative Scr	Measured value
X3	Surgical procedure	0 = guidewire-assisted percutaneous nephrolithotomy, 1 = conventional percutaneous nephrolithotomy
X4	Staghorn calculi	0 = no, 1 = yes
X5	Hydronephrosis	0 = no/ mild, 1 = moderate/ severe
X6	Renal parenchymal thickness	Measured value

**Table 5:** Covariance analysis.

Indicators	VIF	Tolerance
History of diabetes mellitus	5.526	0.181
Preoperative Scr	1.058	0.945
Surgical procedure	1.009	0.991
Staghorn calculi	6.600	0.152
Hydronephrosis	1.017	0.983
Renal parenchymal thickness	1.051	0.952

### 3.6 Multifactorial analysis of bleeding after percutaneous nephrolithotomy

A logistic prediction model was constructed based on univariate analysis, and it was found that preoperative Scr, surgical method, hydronephrosis, and renal parenchyma thickness were all correlates affecting postoperative bleeding after percutaneous nephrolithotomy in patients with upper urinary tract stones (*p* < 0.05, Table 6).

**Table 6:** Multifactorial analysis of bleeding after percutaneous nephrolithotomy.

Factors	Regression coefficient	Standard error	*z*-value	Wald *χ^2^*	*P*-value	OR value	OR value 95% CI
Preoperative Scr	0.210	0.028	7.453	55.552	<0.001	1.233	1.167~1.303
Surgical method	1.045	0.364	2.873	8.256	0.004	2.842	1.394~5.795
Hydronephrosis	0.944	0.351	2.688	7.223	0.007	2.571	1.291~5.119
Renal parenchyma thickness	0.710	0.107	6.645	44.161	<0.001	2.035	1.650~2.509

## 4 Discussion

Guidewire-assisted percutaneous nephrolithotomy combines minimally invasive techniques and interventional therapy to provide a new, efficient, and safe treatment option for patients with upper urinary tract stones. Percutaneous nephrolithotomy enters the kidney through a small incision in the skin with the nephrolithotomy used for direct observation and operation to achieve the therapeutic effect of removing kidney stones. Based on the assistance of the guidewire, doctors can gradually expand the puncture channel with the dilator until it forms a sufficiently large operation channel for smooth entrance of nephrolithotomy and the stone extraction instruments, which indicates that the guidewire-assisted percutaneous nephrolithotomy can accurately locate and operate the stone, being safer and more effective than the conventional percutaneous nephrolithotomy (14, 15). The results showed that the stone removal rate of the guidewire-assisted group was higher than that of the conventional group, while the complication rate, operation time, gastrointestinal function recovery time, hospital days, postoperative bleeding, and hemoglobin drop were lower than that of the conventional group, which meant that the guidewire-assisted percutaneous nephrolithotomy was relatively more effective, especially in improving the postoperative bleeding situation. Even more, 383 patients with upper urinary tract stones were surgically treated in this paper. It was found that 39 patients had severe bleeding of ≥400 mL (an incidence rate of 10.18%) attracted clinical attention. Those risk factors affecting postoperative bleeding should be determined as early as possible, and targeted preventive measures should be taken to reduce the incidence of postoperative bleeding (16, 17).

Scr is produced by muscle creatine phosphokinase catalyzing the breakdown of creatine and released into the blood, which is mainly a measure of renal excretory function. Once renal function is impaired, it can directly reduce the renal filtration and excretory capacity of Scr, and then make the level of Scr rise significantly, which becomes an important indicator for assessing renal function. Clinical studies have confirmed the close relationship between the preoperative Scr level and postoperative bleeding after percutaneous nephrolithotomy. Similarly, our results also found that the preoperative Scr level was higher in the severe bleeding group compared to the mild bleeding group with logistic regression analysis demonstrated that the preoperative Scr was a relevant factor affecting the postoperative bleeding after percutaneous nephrolithotomy, which was mainly due to elevated Scr preoperatively (poor renal function) and lower tolerance for the operation. Moreover, during the operation, it was usually necessary to puncture and establish a channel to crush stones and carry out lithotripsy, especially for those with high preoperative Scr levels, which could further aggravate renal function damage and thus increase the risk of postoperative bleeding (18, 19). In addition, the synthesis and secretion of coagulation factors are reduced when it comes to elevated Scr levels, as the kidney is the main organ for the synthesis and secretion of coagulation factors, which impacts the coagulation function and significantly increases the incidence of postoperative bleeding (20).

Usually during conventional percutaneous nephrolithotomy, doctors need to rely on experience, imaging positioning, and touch to determine the puncture point and channel, and the slightest carelessness may involve renal blood vessels or other important structures being damaged, which leads to an elevated risk of bleeding, whereas guidewire-assisted percutaneous nephrolithotomy via guidewire can provide precise guidance to help the doctor to accurately puncture to the target position under the guidance of ultrasound, indicating that guidewire-assisted can circumvent the risk of blind puncture and significantly reduce the probability of kidney injury and bleeding (21, 22). As seen from the results of this paper, patients who underwent conventional percutaneous nephrolithotomy in the severe bleeding group were significantly more numerous than those in the mild bleeding group, with logistic regression analysis showing that the surgical method was an important factor in postoperative bleeding, implying that postoperative bleeding was more likely to occur in conventional percutaneous nephrolithotomy compared to guidewire-assisted percutaneous nephrolithotomy, in which the guidewire not only provided accurate guidance for the surgeon, but also protected the renal tissues to a certain degree. As for the puncture and dilatation process, the guidewire serves as a safe channel to ensure that the surgical instruments are operated in a precise path, which reduces surgical trauma, shortens the surgical time, effectively avoids unnecessary injuries, and reduces the risk of postoperative hemorrhage (23).

Chen et al. (24) found that hydronephrosis exerted a great influence on the surgical outcome of percutaneous nephrolithotomy through a univariate and multivariate logistic analysis, especially severe hydronephrosis could significantly prolong the operation time and reduce the stone clearance rate. Likely, the results of this paper found that the severity of hydronephrosis in the severe bleeding group was higher than that in the mild bleeding group, confirming that hydronephrosis became one of the factors associated with bleeding after percutaneous nephrolithotomy (25). After analysis, it was concluded that the combination of hydronephrosis in patients with upper urinary tract stones could significantly increase the difficulty and risk of the operation. Normally, in percutaneous nephrolithotomy, doctors need to enter the kidney through a skin puncture to create a working channel to remove the stone, but the occurrence of hydronephrosis leads to changes in the position of the kidney and even causes ambiguity in the anatomical relationship between the kidney and the surrounding tissues, further increasing the risk of bleeding (26, 27). Meanwhile, with the increasing severity of hydronephrosis, the patient’s cortex subsequently becomes weaker with enlarged renal space and more difficulty stone location, which is prone to implicate renal calyces and hemorrhage after damage to the neck of the calyces due to inappropriate manipulation. Beyond that, hydronephrosis can also have a certain impact on the postoperative recovery effect, which directly delays the speed of renal recovery but also increases the incidence of infection, bleeding, and other complications (28).

Studies have indicated (29) that the thicker the renal parenchyma is, the corresponding increase in renal blood supply makes it easier to injure the blood vessels at the junction of the renal cortex and the collecting system during the surgery, which increases the risk of bleeding. During percutaneous nephrolithotomy, if the surgical instrument traverses the renal parenchyma, it can directly damage the rich vascular network, especially the blood vessels at the junction of the renal cortex and the collecting system, which further increases the incidence of bleeding (30). In the present study, the renal parenchyma thickness was higher in the severe bleeding group than in the mild bleeding group, confirming that renal parenchyma thickness became one of the risk factors affecting bleeding after percutaneous nephrolithotomy. Upon analysis, it was detected that with the increase of renal parenchyma thickness, more tissues needed to be traversed during puncture, which increased the risk of vascular damage (31, 32); meantime, the increase in renal parenchyma thickness could lead to the difficulty of selecting the puncture point, which further increased the difficulty of the procedure and the risk of bleeding. In the bargain, the thickness of the renal parenchyma had a greater impact on the surgical field, which made the lithotripsy and stone extraction process more complicated. Therefore, unskilled movements or excessive force during the operation could bring about damage to the renal calyx neck or parenchyma and ultimately lead to hemorrhage (33).

However, there were still some deficiencies in this paper; for example, only a limited number of cases were involved in this study, which could lead to the possibility of some deviations in the results; although multiple risk factors have been explored in this paper, there may still be other factors that had not been taken into account; second, this paper only focused on short-term complications of the patients, coupled with a lack of long-term follow-up data to assess the effectiveness of the surgery. Such being the case, future studies may improve the accuracy and reliability of the study by increasing the number of cases. Further exploration and validation of other possible risk factors can render a more comprehensive assessment of the surgical risk. Additionally, enhancing postoperative follow-up to detect and manage possible complications in a timely manner is expected to improve surgical outcomes.

## 5 Conclusion

In summary, guidewire-assisted percutaneous nephrolithotomy has better efficacy in patients with upper urinary tract stones, which can significantly enhance the stone removal rate and decrease the incidence of complications with a lower recurrence rate. However, there are still some patients suffering from the postoperative bleeding phenomenon, which may be affected by preoperative Scr, surgical procedure, hydronephrosis, renal parenchymal thickness, etc. In this regard, the clinic should formulate corresponding measures for intervention as early as possible to reduce the risk of postoperative bleeding.

## Data Availability

The data analyzed in this study is subject to the following licenses/restrictions: our research is still being further explored by other team members. We will expand the scope of the study and disclose relevant data in future research. The data supporting this paper can be obtained by contacting the corresponding author. Requests to access these datasets should be directed to chenzhong150@163.com.
